# Differential gene expression in mouse primary hepatocytes exposed to the peroxisome proliferator-activated receptor α agonists

**DOI:** 10.1186/1471-2105-7-S2-S18

**Published:** 2006-09-26

**Authors:** Lei Guo, Hong Fang, Jim Collins, Xiao-hui Fan, Stacey Dial, Alex Wong, Kshama Mehta, Ernice Blann, Leming Shi, Weida Tong, Yvonne P Dragan

**Affiliations:** 1 Division of Systems Toxicology, National Center for Toxicological Research, US Food and Drug Administration, Jefferson, AR 72079, USA; 2 Z-Tech Corporation, 3900 NCTR Road, Jefferson, AR 72079, USA; 3 Agilent Technologies, Inc., Santa Clara, CA 95051, USA

## Abstract

**Background:**

Fibrates are a unique hypolipidemic drugs that lower plasma triglyceride and cholesterol levels through their action as peroxisome proliferator-activated receptor alpha (PPARα) agonists. The activation of PPARα leads to a cascade of events that result in the pharmacological (hypolipidemic) and adverse (carcinogenic) effects in rodent liver.

**Results:**

To understand the molecular mechanisms responsible for the pleiotropic effects of PPARα agonists, we treated mouse primary hepatocytes with three PPARα agonists (bezafibrate, fenofibrate, and WY-14,643) at multiple concentrations (0, 10, 30, and 100 μM) for 24 hours. When primary hepatocytes were exposed to these agents, transactivation of PPARα was elevated as measured by luciferase assay. Global gene expression profiles in response to PPARα agonists were obtained by microarray analysis. Among differentially expressed genes (DEGs), there were 4, 8, and 21 genes commonly regulated by bezafibrate, fenofibrate, and WY-14,643 treatments across 3 doses, respectively, in a dose-dependent manner. Treatments with 100 μM of bezafibrate, fenofibrate, and WY-14,643 resulted in 151, 149, and 145 genes altered, respectively. Among them, 121 genes were commonly regulated by at least two drugs. Many genes are involved in fatty acid metabolism including oxidative reaction. Some of the gene changes were associated with production of reactive oxygen species, cell proliferation of peroxisomes, and hepatic disorders. In addition, 11 genes related to the development of liver cancer were observed.

**Conclusion:**

Our results suggest that treatment of PPARα agonists results in the production of oxidative stress and increased peroxisome proliferation, thus providing a better understanding of mechanisms underlying PPARα agonist-induced hepatic disorders and hepatocarcinomas.

## Background

Peroxisome proliferators are structurally diverse chemicals that include industrial pollutants, plasticizers, herbicides, and lipid-lowering drugs. Fibrates including bezafibrate, clofibrate, fenofibrate, WY-14,643, and others are a unique class of hypolipidemic drugs. They function as agonists for peroxisome proliferator-activated receptor alpha (PPARα). PPARα is a transcriptional nuclear receptor and forms a heterodimer with another nuclear receptor, retinoid X receptor (RXR). PPARα/RXR heterodimer binds effectively to the peroxisome proliferator response elements (PPREs) located in promoters of various target genes and regulate the expression of genes involved in lipid metabolism and peroxisome proliferation [1-3]. PPRE consists of direct repeats of TGA/TCCT which is separated by a single nucleotide (DR1) [4]. Fibrates reduce plasma triglyceride and cholesterol levels via the activation of PPARα, which is considered to be the result of induction of fatty acid catabolism in the liver. At the molecular level, fibrates bind to PPARα and increase the expression of genes that involved in fatty acid uptake (fatty acid binding protein, FABP), β-oxidation (acyl-CoA oxidase, ACOX), and ω-oxidation (cytochrome P450) [4-7]. This pharmacological effect of fibrates is responsible for the therapeutic utility, and this effect was observed in preclinical species and also in humans.

Along with the pharmacological effects of fibrates, toxic effects such as marked peroxisome proliferation, hepatomegaly and hepatocarcinoma are observed in rodents [8]. It is accepted that the mode of action (hepatocarcinogenesis) is dependent upon sustained PPARα activation. This mode of action is supported by the observation that even a one year exposure to PPARα agonists was insufficient to cause an increase in the incidence of hepatic neoplasms in PPARα knock-out mice. In addition, peroxisome proliferation and gene expression regulated by PPARα were not remarkably altered. One hypothesis for the carcinogenic mechanism of action of PPARα agonists in rodent liver is based on their ability to elevate peroxisomal β-oxidation and microsomal ω-oxidation of fatty acids, resulting in the generation of hydrogen peroxide. This excess production of hydrogen peroxide results in the generation of reactive oxygen species (ROS) and oxidative stress [9]. The induction of oxidative stress has been suggested as a common pathway for many non-genotoxic carcinogens to elicit their carcinogenicity [10]. In addition, increased peroxisome proliferation in response to activation by PPARα agonists is associated with tumor formation in rodent liver [8]. The combined effect of increased oxidative stress and increased cell proliferation in the rodents exposed to PPARα agonists likely underlies their carcinogenic potential. The precise mechanism of the hepatocarcinogenesis of PPARα agonists in rodents is not fully understood. Since a number of fibrates (e.g., bezafibrate and fenofibrate) are used as therapeutic agents, it is important to analyze the mechanism of liver toxic effects occurred in rodents so that we can better evaluate the safety of these drugs.

Microarray technology provides a comprehensive, rapid and efficient method for large scale profiling of gene expression changes in biological samples (e.g., treatment versus control, disease versus normal). The advantages of DNA microarray technology include the ability to analyze expression patterns of thousands of genes simultaneously. Other advantages include the ability to characterize relationships between genes and the changes in biological processes such as disease states, developmental stages and responses to drugs [10,11]. This method has been successfully employed in identifying gene expression changes in cells, including both hepatic cell lines [12] and isolated hepatocytes [13], in response to various stimuli.

In this study, using mouse primary hepatocytes, we examined global gene expression profiles observed after treatment with several concentrations of three PPARα agonists (i.e. bezafibrate, fenofibrate and WY-14,643). The gene expression profiles showed increased expression of genes involved in fatty acid oxidation and metabolism as expected, and also showed regulation of many genes involved in the production of ROS and those that are associated with liver cancer development. This study also demonstrates the similarity of gene expression changes induced by three different PPARα agonists. In addition, this whole genome microarray analysis performed following in vitro administration of PPARα agonists indicates a plausible mechanism of hepatocarcinogenesis in the mouse liver and may help with the safety assessment of this class of agents.

## Results

### PPARα agonist administration to primary hepatocytes to assess PPARα activity

**Figure 1:**
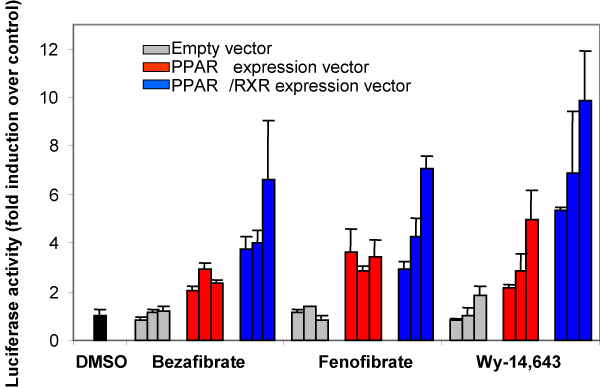
**Activation of PPARα by three PPARα agonists**. Primary hepatocytes were co-transfected with a luciferase reporter construct containing PPRE and with or without PPARα/RXR expression vectors. Twelve hours after transfection, three PPARα agonists, bezafibrate, fenofibrate and WY-14,643 were added at the concentrations as indicated. Cells were harvested after 24 h drug treatment. Luciferase activity was normalized against β-galactosidase activity. The groups having three bars indicate different treatments with the concentrations of 10, 30 and 100 μM, from left to right. Error bars represent standard derivations of two replicates.

In order to estimate whether PPARα activity was elevated by the addition of PPARα agonists, pHD(x3) luciferase plasmid containing three direct tandem copies of a PPRE binding site was used as a reporter plasmid. Primary hepatocytes were co-transfected with pHD(x3) luc reporter plasmid and pSG5-PPARα or pSG5-PPARα/pSG5-RXR expression vectors. Twenty-four hours after transfection, cells were treated with PPARα agonist at various concentrations (10–100 μM) for 24 h. Basal level of luciferase activity was observed (data not shown) when cells were transfected with pHD(x3) luc reporter plasmid and pGS5 empty vector. This could be due to the activity of endogenous PPARα agonists (i.e., fatty acid and their metabolites) [5,14]. Figure 1 shows that mouse primary hepatocytes transfected with pHD(x3) luc reporter plasmid and pGS5 empty vector produced 1.2, 0.8, and 1.8 fold increased in luciferase activity after the treatment with 100 μM bezafibrate, fenofibrate, and WY-14,643, respectively. When cells were treated with the same concentration of drugs in the presence of PPARα expression plasmid, the induction of luciferase activities increased to 2.3, 3.4 and 5.0 fold. Moreover, in the presence of both PPARα and RXR expression plasmids, luciferase activities increased even further to 6.6, 7.1, and 9.9 fold. PPARα activation inductions by these three agonists demonstrate a does-dependent increase for 100 μM compare wtih 10 and 30 μM treatments (Figure 1).

### Gene expression patterns associated with exposures of PPARα agonists

**Figure 2:**
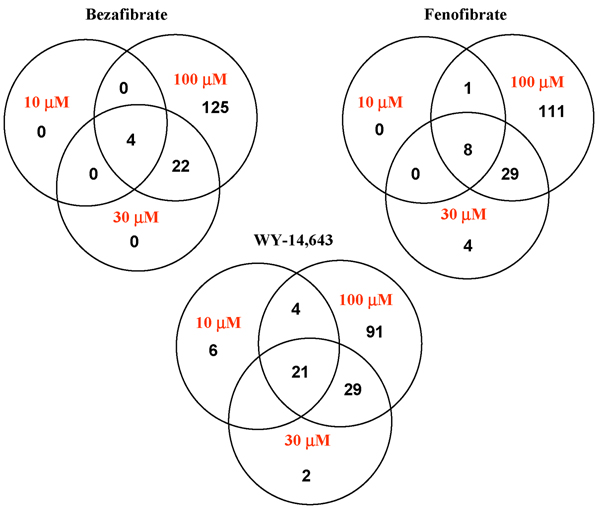
**Numbers of genes regulated by drug treatments**. Numbers of significant genes (FC > 1.5, *P *< 0.05) regulated by bezafibrate, fenofibrate and WY-14,643 at the various concentrations of 10, 30 and 100 μM. Numbers of genes commonly regulated at low, middle and high dose are presented in Venn diagram.

**Figure 3:**
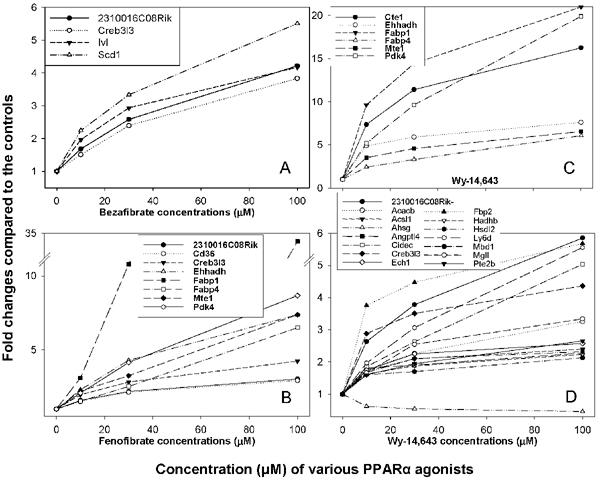
**Dose-dependency of gene expression**. Genes commonly regulated at low, middle and high dose levels were selected.

To understand the molecular mechanisms responsible for the pleiotropic effects of fibrates, mouse primary hepatocytes were treated with three PPARα agonists (Figure 1). RNA was isolated following 24 h treatment and the Agilent Whole Mouse Genome Microarray analysis was performed. Three replicate arrays corresponding to each treatment (10, 30 and 100 μM) of each drug (bezafibrate, fenofibrate and WY-14,643) were compared to the control replicates (DMSO) using Student *t*-test. A gene was considered to be significantly regulated by a drug if the fold change was greater than 1.5 and the *P*-value was less than 0.05. Based on these two criteria, there were 4, 26, and 151 genes that showed an altered expression by the treatments with 10, 30 and 100 μM bezafibrate, respectively. There were 9, 41 and 149 genes altered by 10, 30 and 100 μM fenofibrate, and 31, 52 and 145 genes altered by the treatment with 10, 30 and 100 μM WY-14,643. The Venn diagrams (Figure 2) represent the numbers of differentially expressed genes (DEGs) from three drug treatments at various concentrations. Among these DEGs, 4, 8, and 21 genes were altered in common by bezafibrate, fenofibrate and WY-14,643 treatments across 3 doses, respectively (Figure 2). These commonly regulated genes showed clear dose-dependent changes (Figure 3). Except for the down regulation of Ahsg by WY-14,643, all genes were identified as up-regulated. Expression of Pdk4 and Cte1 showed a particularly prominent induction (10–20 fold at 100 μM) by treatment of WY-14,643 (Fig. 3C), as did Fabp1 with 20–30 fold induction by 100 μM treatments of fenofibrate and WY-14,643 (Figs. 3B &3C).

**Figure 4:**
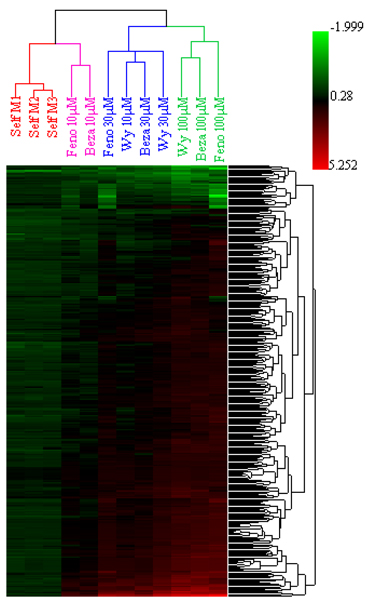
Two-dimensional hierarchical cluster analysis (HCA) of significant genes induced by bezafibrate, fenofibrate and WY-14,643.

**Figure 5:**
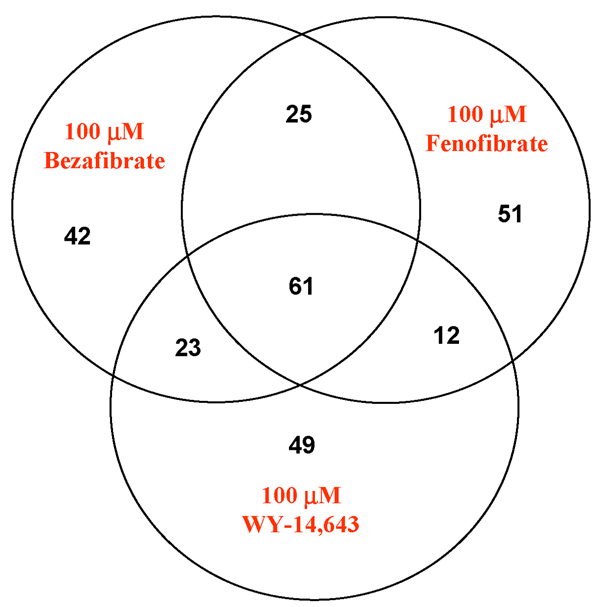
Distribution and overlap of significant genes (fc > 1.5 and *p *< 0.05) among bezafibrate fenofibrate and WY-14,643 treatments of 100 μM.

Hierarchical clustering analysis with DEGs revealed that these genes were grouped together based on treatment doses rather than on specific drugs (Figure 4), indicating that a class effect was detectable. Taking into consideration the fact that most of the genes altered by low (10 μM) and middle (30 μM) dose treatments were also altered by high (100 μM) dose treatments, further analysis was focused on the genes regulated by the high dose treatments. Figure 5 represents the numbers of genes regulated by the 100 μM treatments of three drugs and the numbers of overlapping genes among three drug treatments. Treatments with 100 μM of bezafibrate, fenofibrate and WY-14,643 resulted in 151, 149 and 145 genes with altered expression, respectively. Among them, 61 genes were concordantly regulated by three drugs and 121 genes were regulated commonly by at least 2 drugs. The correlation of log2 fold changes based on 61 genes which were regulated commonly by three drugs was determined by pairwise comparisons (Table 1). Comparison of gene expression profiles resulted in relatively high correlations (0.94–0.97) between three drug treatments, indicating that gene expression patterns are identical at the 100 μM concentration despite any differences among the drugs.

### Gene function analysis

Using Ingenuity Pathway Analysis, we conducted gene function analysis with these 121 commonly regulated genes. Not surprisingly, the largest categories of induced genes were those involved in lipid metabolism (49 genes), including oxidation, modification, and metabolism. Table 2 shows the genes involved in oxidation (19 genes), of which 10 were involved in β-oxidation. As expected, many genes directly regulated by PPARα including Cpt1 (carnitine palmitoyltransferase 1), Fabp1 (fatty acid binding protein 1), Acox1 (acyl-Coenzyme A oxidase 1, palmitoyl), and Ehhadh (enoyl-Coenzyme A, hydratase/3-hydroxyacyl Coenzyme A dehydrogenase) were identified in this study. Induction of these genes corroborates previous data, and also serves as the validation of our microarray experiments. Several novel genes including Acacb, Acsl1, Ech1, Hadhb, and Pdk4 that are involved in oxidation of lipid metabolism were responsive to the treatments of PPARα agonists. In addition, four genes (Aldh3a2, Apoc2, Cd36, and Slc25a10) associated with the production of ROS exhibited 1.5–3.0 fold up-regulation, and two genes (Acox1 and Pex11a) involved in the cellular proliferation of peroxisomes were 3-fold up-regulated.

Administration of fibrates to rodents results in hepatic diseases and hepatocarcinoma [8]. For gene function analysis, we also concentrated on genes involved in these effects. There were six genes related to hepatic disorders (Table 3), and all were up-regulated by fibrate administration. Four of these genes are involved in oxidation of lipids. In addition, 11 genes classified as being related to the development of liver cancer had altered expression levels. These include liver fatty acid binding protein 1 (Fabp1), lymphocyte antigen 6 complex locus D (Ly6d), monoglyceride lipase (Mgll), and angiopoietin-like 4 (Angptl4), with a wide range of up-regulation (1.6–34.4 fold) after treatments with these three PPARα agonists.

## Discussion

Fibrates, members of peroxisome proliferators and agonists of PPARα, are used to treat hyperlipidemia by reducing plasma triglyceride and cholesterol levels via accelerating lipid metabolism. In rodents, administration of fibrates can induce hepatomegaly and hepatocarcinoma, possibly due to the induction of cell proliferation and increased oxidative stress. Examination of PPARα-deficient mice demonstrated that the activation of PPARα is required exclusively for mediating both pharmacological (hypolipidemic) and toxic (carcinogenic) responses of fibrate administration [15,16]. However, mechanisms of fibrate-induced hepatocarcinoma development and the potential risk of use of these drugs to humans remain unclear. Examination of gene expression profiles is an important approach that may help us better understand PPARα-mediated pleiotropic effects.

In this study, microarray analysis was applied to generate a molecular portrait of gene expression in mouse primary hepatocytes exposed to fibrates (Figure 1). We treated mouse primary hepatocytes with three fibrates (bezafibrate, fenofibrate and WY-14,643) at multiple doses (0, 10, 30, and 100 μM). Although global gene analysis study was conducted in vitro [17], the design of this study (i.e., treatment with multiple fibrates at multiple level doses) permitted us to detect whether changes in gene expressions are a class effect (e.g., genes are commonly regulated by multiple drugs) and whether changes are dose-dependent as well. Indeed, the majority of genes regulated by low and middle doses were also identified in high dose treatments. For example, 4/4, 9/9, and 25/31 genes that were regulated by 10 μM treatments of bezafibrate, fenofibrate and WY-14,643, respectively, were also found to be regulated in 100 μM treatments (Figure 2). In addition, dose-response dependency in gene expressions was also observed for the genes commonly regulated at multiple doses (Figure 3). The dose-dependent expression levels of genes altered by PPARα agonists allowed us to assess biological activity of this class of agents.

PPARα agonists have a therapeutic role in the management of fatty acid metabolism through their effects on β-oxidation and lipid transport. The gene expression changes in common across PPARα agonists may indicate those genes are directly regulated by PPARα stimulation. In this study, we demonstrated that 121 DEGs were altered in common by at least two of the three PPARα agonists tested. The Ingenuity Pathway Analysis was used to analyze gene functions and to provide pathway annotations. Based on this analysis, many of these genes (49 genes) are involved in the oxidation of fatty acids (Table 2) as has been previously shown for this class of agents [4,18]. For example, acyl-coA synthetase catalyzes the precursor step to β-oxidation (ligates CoA to a free fatty acid) and three members of the long chain acyl CoA synthestase family (Acsl1, Acsl4, and Acsl5) were increased. This observation is supported by the work of Schoonjans et al. who demonstrated that the expression of Acs is altered by fibrates and that there is a PPRE in the Acsl promoter [19]. These findings also agree with those of Cornwall et al. who reported that the expression of Acsl was elevated in the liver of rats exposed to fenofibrate [20]. The effects on the β-oxidation pathway also include the induction of the first enzyme of peroxisomal β-oxidation, acyl-CoA oxidase (Acox), as well as the next enzyme in the cascade, enoyl-CoA hydratase/3-hydroxyacyl-CoA dehydrogenase (Ehhadh). The identification of a large number of lipid metabolizing genes following exposure to several PPARα agonists is in concordance with the known biochemical and molecular effects of these hypolipidemic agents to regulate lipid metabolism.

PPARα agonists are also considered to be nongenotoxic carcinogens in rodents. Oxidative stress has been proposed as a common pathway for many non-genotoxic carcinogens [21]. In the present study, 10 genes involved in fatty acid β-oxidation were up-regulated upon exposure to PPARα agonists (Table 2), which included Acox, the key enzyme of peroxisomal fatty acid β-oxidation system. The elevation of peroxisomal fatty acid β-oxidation such as occurs with PPARα agonist exposure in rodents results in the elevated generation of hydrogen peroxide [9]. Substantial production of hydrogen peroxide causes oxidative stress and the induction of ROS. The increased ROS associated with elevated levels of Acox has been postulated to mediate the hepatocarcinogenesis resulting from PPARα exposure in rodents. We observed four genes (Aldh3a2, Apoc2, Cd36, and Slc25a10) associated with the production of ROS were up-regulated. A growing body of evidence indicates that Cd36 (CD36 antigen) is involved in the cytotoxicity associated with inflammation and is an essential mediator of the production of ROS [22]. In addition, six genes classified as related to hepatic disorders were identified as being up-regulated (Table 3). These observations support the hypothesis that increased peroxisome proliferation results in oxidative stress, which may be due to the disproportionate increase in the level of oxidation versus antioxidation enzyme activities [8].

It is believed that the activation of PPARα and ensuing cascade effects are linked to both pharmacological and tumorigenic effects of PPARα agnoists [8]. The carcinogenic response seems likely to be associated with both the induction of oxidative stress and the increased cell proliferation from peroxisome proliferation after treatment with these chemicals. In this study, we found that two genes (Acox1 and Pex11a) associated with cellular proliferation of peroxisomes were up-regulated about 3-fold. The level of peroxisomal biogenesis factor 11 (Pex11) correlates roughly with peroxisome abundance in the cell, and over-expression of Pex11 alone is sufficient to accelerate peroxisome division and to increase peroxisome abundance [23]. It is thought that alteration in the balance between cell proliferation and apoptosis is causally related to the induction of liver tumors, and induced cell proliferation plays a key role in carcinogenesis in animals and humans [24,25].

Based on the Ingenuity Pathway Analysis, 11 genes associated with liver cancer development were up-regulated by at least two PPARα agonists tested (Table 3). For example, Bnip3 (BCL2 19 kDa-interacting protein 1), a pro-apoptotic factors of the Bcl-2-family, has been previously shown to be up-regulated in malignant tumors [26]. Diazepam binding inhibitor (Dbi), interacts with hepatocyte nuclear factor-4 α that transcriptionally regulates the genes involved in both lipid and glucose metabolism [27], was also increased. Previous studies indicated that Dbi levels are higher in hepatocellular carcinoma (HCC) patients [28], and the elevation of Dbi expression is useful in evaluating malignancy and in diagnostic approaches of tumors in liver tissue [29]. Fatty acid-binding proteins (Fabps) are involved in lipid metabolism by intracellular transport of long-chain fatty acids. Liver fatty acid-binding protein (Fabp1) is demonstrated immunohistochemically in human hepatocellular malignancies, suggesting that its immunoreactivity is a candidate for the tumor marker in hepatic cell malignancies [30]. Fatty acid synthase (Fasn) is the key enzyme of *de novo *fatty acid synthesis. The over-expression of Fasn is an early phenomenon presented in both hormonally and chemically induced rat hepatocarcinogenesis [31]. Hypoxia inducible factor-1 alpha (Hif1a) regulates the expression of a myriad of genes involved in oxygen transport, glucose uptake, glycolysis and angiogenesis. The expression of Hif1a in HCC tissue is higher than that in paraneoplastic tissue or normal liver tissue, and Hif1a plays an important role in neovascularization in HCC [32]. Lgals3 (lectin) has been demonstrated to be associated with assorted processes such as cell growth, tumor transformation and metastasis. It has been reported that Lgals3 expression was induced in cirrhotic liver and HCC, and that the expression of Lgals3 in proliferating cells possibly indicates an early neoplastic event [33]. The plasminogen activation system, including PAI-1 (plasminogen activator inhibitor 1), plays a crucial role in the process of cancer. PAI-1 is increased in HCC, and its expression is related to the invasiveness, metastasis, and prognosis [34,35]. Our findings support the observation that PPARα agonists increase proliferation of peroxisomes in rodent hepatocytes and alter lipid metabolism. In addition, the gene expression profiles indicate a number of leads toward understanding PPARα agonist-induced hepatocarcinogenesis in the mouse.

## Conclusion

In summary, primary mouse hepatocytes were treated with various concentrations (10, 30, 100 μM) of three PPARα agonists (bezafibrate, fenofibrate, and WY-14,643) for 24 hr. Transactivation analysis indicated that these three agents activated the PPARα in a dose-dependent manner. Global gene expression analysis was performed on whole mouse genome arrays following exposure of the mouse hepatocyte cultures to PPARα agonists. Hierarchal clustering analysis of these gene expression profiles indicated that expression profiles of DEGs were clustered based on doses rather than specific drugs, indicating there is a common effect across this class of compound. Gene expression changes were detected in mouse hepatocytes following exposure to 100 μM bezafibrate (151 genes), fenofibrate (149 genes), and WY-14,643 (145 genes). The expression of 121 genes was changed in common by at least two of the PPARα agonists tested. Based on Ingenuity Pathway Analysis, many of these genes (49) function in lipid metabolism. An additional 11 genes were mapped to cancer associated functions. Clear dose-dependent changes in DEGs were determined based on magnitude of fold change. These results provide a better understanding of the underlying mechanisms of the hepatic effects of PPARα agonists in the mouse.

## Materials and methods

### Chemicals and cell treatments

Bezafibrate and fenofibrate were purchased from Sigma (St. Louis, MO). WY-14,643 was purchased from Chemsyn Science Laboratories (Lenexa, KS). All compounds were prepared as 1000 x stock solutions in dimethyl sulfoxide (DMSO) and added to cell cultures in final concentrations of 10–100 μM. The same amount of DMSO (0.1% v/v) was added to control cells. During the treatments, serum-free medium was used with supplements (see below).

Three 6–8 week-old C57/BL6 male mice were obtained from the breeding colony of the FDA's National Center for Toxicological Research. Mice were anesthetized with 1.5 ml/kg of nembutal sodium solution containing 50 mg/ml of pentobarbital sodium prior to undergoing liver perfusion. All animals used in this study were handled in accordance with the principles and guidelines prepared by the National Institutes of Health, USA. Mouse primary hepatocytes were isolated by a two-stage collagenase perfusion process according to the methods described by Seglen et al. and Kreamer et al. [36,37]. Primary hepatocytes were suspended in L-15 medium containing 2 mg/ml BSA, 18 mM HEPES, 3 mg/ml proline, 1 mg/ml galactose, 0.1% insulin-transferrin-selenite, 10 ng/ml epidermal growth factor, 50 U/ml penicillin, and 50 μg/ml streptomycin. The cells were treated with drugs six hours after plating.

### Plasmid transfection and reporter assays

Luciferase reporter plasmid, pHD(x3) luc, contains three direct tandem copies of PPRE binding site, was used as previously described [38]. pSG5-PPARα and pSG5-RXR expression plasmids were obtained from Dr. Marek Michalak (University of Alberta, Canada). Plasmid DNAs were purified by column chromatography (Qiagen Inc., Valencia, CA). Primary hepatocytes were plated in L15 medium at 1 × 10^5 ^cells/per well for 6 wells, and transfections were carried out after cell attachment with FuGENE reagent (Roche Diagnostics, Indianapolis, IN). Briefly, 300 μl of L15 (no additives) containing 9 μl of FuGENE reagent was mixed with a total of 3 μg of plasmid DNA with or without pSG5-PPARα/pSG5-RXR, luciferase reporter plasmid, and pSVβ-gal (internal control). This mixture was added to cells for a 10 min incubation at room temperature. For induction, medium was replaced with fresh L15 without BSA after 12 h incubation with plasmids/FuGENE. The PPARα agonists were added for 24 h at appropriate concentrations. To harvest lysates for luciferase activity, hepatocytes were washed twice in PBS, and then lysed in 150 μl of 1x reporter lysis buffer (Promega Corp., Madison, WI). Luciferase activity was measured using the Luciferase Assay System (Promega). Luminescence was determined using an automatic luminometer, LumiTeum II (Harta Instruments, Gaithersburg, MD). β-galactosidase enzyme assay was carried out using β-galactosidase Enzyme Assay System (Promega). Luciferase activity was normalized against β-galactosidase activity from the same lysate. Each assay was performed in duplicates.

### RNA isolation and quality control

Total RNA from cells was isolated using an RNeasy system (Qiagen). The yield of the extracted RNA was determined spectrophotometrically by measuring the optical density at 260 nm. The purity and quality of extracted RNA were evaluated using the RNA 6000 LabChip and Agilent 2100 Bioanalyzer (Agilent Technologies, Santa Clara, CA). Only high quality RNA with RNA integrity numbers (RINs) greater than 7.5 were used for microarray experiments.

### Preparation of labeled in vitro transcribed cRNA targets

All total RNA samples were labeled by direct incorporation of cyanine 3 or cyanine 5 dyes using the Agilent Low RNA Input Linear Amplification Kit (Santa Clara, CA). A 500 ng quantity of total RNA was input to each reaction. Labeled cRNAs were purified using the Qiagen RNeasy Mini kit, and were analyzed for quality and quantity using standard UV spectrometry and the Agilent Bioanalyzer.

### Hybridization of labeled cRNA to microarrays and microarray imaging

Cyanine 3 labeled cRNAs were mixed with cyanine 5 labeled cRNAs for hybridization to microarrays. Each test sample was hybridized against its corresponding control sample to two microarrays in a dye-swapped pair. Agilent Whole Mouse Genome Microarrays (Santa Clara, CA) were hybridized using the Agilent Gene Expression Hybridization kit and washed using Agilent Gene Expression Wash Buffers according to the manufacturers' protocols. Hybridized microarrays were scanned using the Agilent DNA Microarray Scanner and data were extracted from images using the Agilent Feature Extraction (version 7.5) software using default settings.

### Microarray data analysis

The Agilent Whole Mouse Genome Microarray that contains 43,790 probes was used to generate gene expression profiles for bezafibrate, fenofibrate, and WY-14,643 at three dose levels (10 μM, 30 μM and 100 μM) with three biological replicates (hepatocytes isolated from mouse A, B, C). Each treated sample was paired with a control (non-treated hepatocytes) using a dye swap experiment design, resulting in 54 arrays [3 chemicals × 3 doses × 3 animals × (2 dye swap)]. In addition, self-self hybridizations were also conducted for each of three controls with three technical replicates, resulting in nine additional arrays. In total, 63 hybridizations were performed for this study.

Linear & Lowess method consists of median scaling to 1000 for each channel per array with a follow up Lowess normalization. The parameters used in Lowess normalization were: smoothing factor = 0.2 and robustness iterations = 3. Low intensity (<500) spots were filtered out after normalization and a subset of 25,010 genes was generated for further data analysis. The DEGs were identified using a combination of Student *t*-test and fold change (FC). A gene was considered differentially expressed if *P*-value was less than 0.05 and the FC was greater than 1.5. Two lists of DEGs were obtained. One list was obtained by comparing the nine self-self hybridization arrays with those polarity+ arrays in which control and treatment samples were labeled with Cy3 and Cy5, respectively. The other list was obtained by comparing the nine self-self hybridization arrays with those polarity- arrays in which control and treatment samples were labeled with Cy5 and Cy3, respectively. The genes in common between these two lists of DEGs were considered as final DEGs and used for biological interpretation. The described analysis approach, in terms of self-self hybridization experiment design, will be reported somewhere else, where its comparative performance analysis was conducted.

Microarray data management and analysis were conducted using an FDA microarray software, ArrayTrack [39,40]. ArrayTrack also provides functionality for the interpretation of gene expression data. For example, pathway analysis is based on the Pathway Library in ArrayTrack, which contains pathways from Kyoto Encyclopedia of Genes and Genomes (KEGG) [41] and PathArt (Jubilant Biosys Ltd., Columbia, MD). The Fisher Exact Test [42] is implemented in ArrayTrack to assess the statistical significance of identified pathways. Ingenuity Pathway Analysis software (Mountain View, CA) was also used for gene function and pathway analysis. S-Plus (Insightful Corp., Seattle, WA) was used in this study for the statistical calculation.

## Authors' contributions

LG performed the analysis of microarray data and wrote the manuscript. HF, XHF and WT performed data analysis. LS and YD helped writing the manuscript. JC, AW, KM conducted the microarray experiment and generated the raw data. SD and EB performed hepatocytes isolation and RNA extraction.

## Figures and Tables

**Table 1:** Correlation coefficients of Log2 FC for 100 μM treatments with different PPARα agonists

	Bezafibrate	Fenofibrate	WY-14,643
Bezafibrate	1.0000	0.9652	0.9642
Fenofibrate	0.9652	1.0000	0.9419
WY-14,643	0.9642	0.9419	1.0000

**Table 2:** Genes involved in oxidation in mouse primary hepatocytes treated with 100 μM of 3 drugs for 24 hours

**Gene symbol**	**Gene description**	**Locus link ID**	**Bezafibrate**		**Fenofibrate**		**WY-14,643**	
			
			**fc**	***p***	**fc**	***p***	**fc**	***p***
*Abcd3	ATP-binding cassette, sub-family D (ALD), member 3	19299			1.88	0.032	1.97	0.017
Acacb	acetyl-Coenzyme A carboxylase beta	100705	2.99	0.000	2.85	0.009	3.26	0.008
*Acadvl	acyl-Coenzyme A dehydrogenase, very long chain	11370	1.97	0.000	2.11	0.017		
*Acox1	acyl-Coenzyme A oxidase 1, palmitoyl	11430	2.91	0.019	3.01	0.012		
Acsl1	acyl-CoA synthetase long-chain family member 1	14081	1.92	0.033	2.04	0.013	2.40	0.001
Acsl4	acyl-CoA synthetase long-chain family member 4	50790	1.75	0.005	1.91	0.012	1.66	0.019
Acsl5	acyl-CoA synthetase long-chain family member 5	433256	1.67	0.010	1.57	0.018	1.55	0.001
Aldh1a2	aldehyde dehydrogenase family 1, subfamily A2	19378	2.13	0.013	2.12	0.043		
Cd36	CD36 antigen	12491	3.00	0.017	2.93	0.032		
*Cpt1a	carnitine palmitoyltransferase 1a, liver	12894			1.52	0.000	1.61	0.015
*Ech1	enoyl coenzyme A hydratase 1, peroxisomal	51798	2.79	0.011	2.64	0.007	2.57	0.005
*Ehhadh	enoyl-Coenzyme A, hydratase/3-hydroxyacyl Coenzyme A dehydrogenase	74147	7.12	0.000	7.38	0.001	7.61	0.004
Fabp1	fatty acid binding protein 1, liver	14080	27.80	0.001	34.35	0.004	20.96	0.014
*Hadha	hydroxyacyl-Coenzyme A dehydrogenase/3-ketoacyl-Coenzyme A thiolase/enoyl-Coenzyme A hydratase (trifunctional protein), alpha subunit	97212	1.95	0.035	1.81	0.002	1.92	0.000
*Hadhb	hydroxyacyl-Coenzyme A dehydrogenase/3-ketoacyl-Coenzyme A thiolase/enoyl-Coenzyme A hydratase (trifunctional protein), beta subunit	231086	2.24	0.020	2.18	0.004	2.24	0.007
*Hsd17b4	hydroxysteroid (17-beta) dehydrogenase 4	15488	1.53	0.023	1.67	0.012		
Pdk4	pyruvate dehydrogenase kinase, isoenzyme 4	27273	10.18	0.012	8.68	0.004	19.88	0.000
Scd	stearoyl-Coenzyme A desaturase 1	20249	5.50	0.004	4.51	0.023		
*Slc27a2	solute carrier family 27 (fatty acid transporter), member 2	26458	2.79	0.019	2.98	0.026		

*Ten genes also involved in β-oxidation. fc, Fold change; *p*, *P*-value.

**Table 3:** Genes associated with hepatic diseases and liver cancer development in mouse primary hepatocytes treated with 100 μM of 3 drugs for 24 hours (based on Ingenuity Pathway Analysis)

**Gene symbol**	**Gene description**	**Locus link ID**	**Beza**		**Feno**		**WY**	
			
			**fc**	***p***	**fc**	***p***	**fc**	***p***
*Hepatic diseases*								
Abcb4	ATP-binding cassette, sub-family B (MDR/TAP), member 4	18670	1.72	0.006	2.06	0.029		
Acox1	acyl-Coenzyme A oxidase 1, palmitoyl	11430	2.91	0.019	3.01	0.012		
Fabp1	fatty acid binding protein 1, liver	14080	27.80	0.001	34.35	0.004	20.96	0.014
Hadha	hydroxyacyl-Coenzyme A dehydrogenase/3-ketoacyl-Coenzyme A thiolase/enoyl-Coenzyme A hydratase (trifunctional protein), alpha subunit	97212	1.95	0.035	1.81	0.002	1.92	0.000
Insig1	insulin induced gene 1	231070	1.68	0.001			1.64	0.000
Scd	stearoyl-Coenzyme A desaturase 1	20249	5.50	0.004	4.51	0.023		
*Liver cancer development*								
Angptl4	angiopoietin-like 4	57875	2.24	0.009	1.87	0.017	2.28	0.011
Bnip3	BCL2 19kDa-interacting protein 1	12176	1.97	0.014			2.31	0.036
Dbi	diazepam binding inhibitor	13167	1.88	0.006	1.82	0.005	1.68	0.035
Fabp1	fatty acid binding protein 1, liver	14080	27.80	0.001	34.35	0.004	20.96	0.014
Fabp2	fatty acid binding protein 2, intestinal	14079	2.88	0.020	2.91	0.014		
Fasn	fatty acid synthase	14104	2.03	0.014	1.89	0.015	1.72	0.000
Hif1a	hypoxia inducible factor 1, alpha subunit	15251			1.70	0.012	1.64	0.033
Lgals3	lectin, galactose binding, soluble 3	16854	1.89	0.023			2.05	0.000
Ly6d	lymphocyte antigen 6 complex, locus D	17068	5.21	0.012	4.23	0.000	5.56	0.000
Mgll	monoglyceride lipase	23945	3.51	0.028	3.78	0.013	3.34	0.008
Serpine1	serine peptidase inhibitor, clade E, mem1	18787	2.35	0.016			2.52	0.013

(fc, Fold change; *p*, *P*-value; Beza, bezafibrate; Feno, fenofibrate; WY, WY-14,643)

## References

[B1] Schoonjans K, Staels B, Auwerx J (1996). Role of the peroxisome proliferator-activated receptor (PPAR) in mediating the effects of fibrates and fatty acids on gene expression. J Lipid Res.

[B2] Schoonjans K, Staels B, Auwerx J (1996). The peroxisome proliferator activated receptors (PPARS) and their effects on lipid metabolism and adipocyte differentiation. Biochim Biophys Acta.

[B3] Corton JC, Anderson SP, Stauber A (2000). Central role of peroxisome proliferator-activated receptors in the actions of peroxisome proliferators. Annu Rev Pharmacol Toxicol.

[B4] Tugwood JD, Issemann I, Anderson RG, Bundell KR, McPheat WL, Green S (1992). The mouse peroxisome proliferator activated receptor recognizes a response element in the 5' flanking sequence of the rat acyl CoA oxidase gene. Embo J.

[B5] Dreyer C, Krey G, Keller H, Givel F, Helftenbein G, Wahli W (1992). Control of the peroxisomal beta-oxidation pathway by a novel family of nuclear hormone receptors. Cell.

[B6] Muerhoff AS, Griffin KJ, Johnson EF (1992). The peroxisome proliferator-activated receptor mediates the induction of CYP4A6, a cytochrome P450 fatty acid omega-hydroxylase, by clofibric acid. J Biol Chem.

[B7] Vanden Heuvel JP, Sterchele PF, Nesbit DJ, Peterson RE (1993). Coordinate induction of acyl-CoA binding protein, fatty acid binding protein and peroxisomal beta-oxidation by peroxisome proliferators. Biochim Biophys Acta.

[B8] Klaunig JE, Babich MA, Baetcke KP, Cook JC, Corton JC, David RM, DeLuca JG, Lai DY, McKee RH, Peters JM (2003). PPARalpha agonist-induced rodent tumors: modes of action and human relevance. Crit Rev Toxicol.

[B9] Yeldandi AV, Rao MS, Reddy JK (2000). Hydrogen peroxide generation in peroxisome proliferator-induced oncogenesis. Mutat Res.

[B10] Schena M, Shalon D, Davis RW, Brown PO (1995). Quantitative monitoring of gene expression patterns with a complementary DNA microarray. Science.

[B11] Lipshutz RJ, Fodor SP, Gingeras TR, Lockhart DJ (1999). High density synthetic oligonucleotide arrays. Nat Genet.

[B12] Burczynski ME, McMillian M, Ciervo J, Li L, Parker JB, Dunn RT, Hicken S, Farr S, Johnson MD (2000). Toxicogenomics-based discrimination of toxic mechanism in HepG2 human hepatoma cells. Toxicol Sci.

[B13] Waring JF, Ciurlionis R, Jolly RA, Heindel M, Ulrich RG (2001). Microarray analysis of hepatotoxins in vitro reveals a correlation between gene expression profiles and mechanisms of toxicity. Toxicol Lett.

[B14] Issemann I, Green S (1990). Activation of a member of the steroid hormone receptor superfamily by peroxisome proliferators. Nature.

[B15] Lee SS, Pineau T, Drago J, Lee EJ, Owens JW, Kroetz DL, Fernandez-Salguero PM, Westphal H, Gonzalez FJ (1995). Targeted disruption of the alpha isoform of the peroxisome proliferator-activated receptor gene in mice results in abolishment of the pleiotropic effects of peroxisome proliferators. Mol Cell Biol.

[B16] Peters JM, Aoyama T, Cattley RC, Nobumitsu U, Hashimoto T, Gonzalez FJ (1998). Role of peroxisome proliferator-activated receptor alpha in altered cell cycle regulation in mouse liver. Carcinogenesis.

[B17] Vanden Heuvel JP, Kreder D, Belda B, Hannon DB, Nugent CA, Burns KA, Taylor MJ (2003). Comprehensive analysis of gene expression in rat and human hepatoma cells exposed to the peroxisome proliferator WY14,643. Toxicol Appl Pharmacol.

[B18] Bardot O, Aldridge TC, Latruffe N, Green S (1993). PPAR-RXR heterodimer activates a peroxisome proliferator response element upstream of the bifunctional enzyme gene. Biochem Biophys Res Commun.

[B19] Schoonjans K, Watanabe M, Suzuki H, Mahfoudi A, Krey G, Wahli W, Grimaldi P, Staels B, Yamamoto T, Auwerx J (1995). Induction of the acyl-coenzyme A synthetase gene by fibrates and fatty acids is mediated by a peroxisome proliferator response element in the C promoter. J Biol Chem.

[B20] Cornwell PD, De Souza AT, Ulrich RG (2004). Profiling of hepatic gene expression in rats treated with fibric acid analogs. Mutat Res.

[B21] Klaunig JE, Xu Y, Isenberg JS, Bachowski S, Kolaja KL, Jiang J, Stevenson DE, Walborg EF (1998). The role of oxidative stress in chemical carcinogenesis. Environ Health Perspect.

[B22] Cho S, Park EM, Febbraio M, Anrather J, Park L, Racchumi G, Silverstein RL, Iadecola C (2005). The class B scavenger receptor CD36 mediates free radical production and tissue injury in cerebral ischemia. J Neurosci.

[B23] Li X, Gould SJ (2003). The dynamin-like GTPase DLP1 is essential for peroxisome division and is recruited to peroxisomes in part by PEX11. J Biol Chem.

[B24] Khan SH, Sorof S (1994). Liver fatty acid-binding protein: specific mediator of the mitogenesis induced by two classes of carcinogenic peroxisome proliferators. Proc Natl Acad Sci USA.

[B25] Roberts RA, James NH, Hasmall SC, Holden PR, Lambe K, Macdonald N, West D, Woodyatt NJ, Whitcome D (2000). Apoptosis and proliferation in nongenotoxic carcinogenesis: species differences and role of PPARalpha. Toxicol Lett.

[B26] Stepan H, Leo C, Purz S, Hockel M, Horn LC (2005). Placental localization and expression of the cell death factors BNip3 and Nix in preeclampsia, intrauterine growth retardation and HELLP syndrome. Eur J Obstet Gynecol Reprod Biol.

[B27] Petrescu AD, Payne HR, Boedecker A, Chao H, Hertz R, Bar-Tana J, Schroeder F, Kier AB (2003). Physical and functional interaction of Acyl-CoA-binding protein with hepatocyte nuclear factor-4 alpha. J Biol Chem.

[B28] Venturini I, Zeneroli ML, Corsi L, Baraldi C, Ferrarese C, Pecora N, Frigo M, Alho H, Farina F, Baraldi M (1998). Diazepam binding inhibitor and total cholesterol plasma levels in cirrhosis and hepatocellular carcinoma. Regul Pept.

[B29] Venturini I, Alho H, Podkletnova I, Corsi L, Rybnikova E, Pellicci R, Baraldi M, Pelto-Huikko M, Helen P, Zeneroli ML (1999). Increased expression of peripheral benzodiazepine receptors and diazepam binding inhibitor in human tumors sited in the liver. Life Sci.

[B30] Suzuki T, Watanabe K, Ono T (1990). Immunohistochemical demonstration of liver fatty acid-binding protein in human hepatocellular malignancies. J Pathol.

[B31] Evert M, Schneider-Stock R, Dombrowski F (2005). Overexpression of fatty acid synthase in chemically and hormonally induced hepatocarcinogenesis of the rat. Lab Invest.

[B32] Huang GW, Yang LY, Lu WQ (2005). Expression of hypoxia-inducible factor 1alpha and vascular endothelial growth factor in hepatocellular carcinoma: Impact on neovascularization and survival. World J Gastroenterol.

[B33] Hsu DK, Dowling CA, Jeng KC, Chen JT, Yang RY, Liu FT (1999). Galectin-3 expression is induced in cirrhotic liver and hepatocellular carcinoma. Int J Cancer.

[B34] Itoh T, Hayashi Y, Kanamaru T, Morita Y, Suzuki S, Wang W, Zhou L, Rui JA, Yamamoto M, Kuroda Y (2000). Clinical significance of urokinase-type plasminogen activator activity in hepatocellular carcinoma. J Gastroenterol Hepatol.

[B35] Zheng Q, Tang ZY, Xue Q, Shi DR, Song HY, Tang HB (2000). Invasion and metastasis of hepatocellular carcinoma in relation to urokinase-type plasminogen activator, its receptor and inhibitor. J Cancer Res Clin Oncol.

[B36] Kreamer BL, Staecker JL, Sawada N, Sattler GL, Hsia MT, Pitot HC (1986). Use of a low-speed, iso-density percoll centrifugation method to increase the viability of isolated rat hepatocyte preparations. In Vitro Cell Dev Biol.

[B37] Seglen PO (1976). Preparation of isolated rat liver cells. Methods Cell Biol.

[B38] Zhang B, Marcus SL, Miyata KS, Subramani S, Capone JP, Rachubinski RA (1993). Characterization of protein-DNA interactions within the peroxisome proliferator-responsive element of the rat hydratase-dehydrogenase gene. J Biol Chem.

[B39] Tong W, Harris S, Cao X, Fang H, Shi L, Sun H, Fuscoe J, Harris A, Hong H, Xie Q (2004). Development of public toxicogenomics software for microarray data management and analysis. Mutat Res.

[B40] Tong W, Cao X, Harris S, Sun H, Fang H, Fuscoe J, Harris A, Hong H, Xie Q, Perkins R (2003). ArrayTrack--supporting toxicogenomic research at the U.S. Food and Drug Administration National Center for Toxicological Research. Environ Health Perspect.

[B41] Kanehisa M, Goto S, Kawashima S, Nakaya A (2002). The KEGG databases at GenomeNet. Nucleic Acids Res.

[B42] Zeeberg BR, Feng W, Wang G, Wang MD, Fojo AT, Sunshine M, Narasimhan S, Kane DW, Reinhold WC, Lababidi S (2003). GoMiner: a resource for biological interpretation of genomic and proteomic data. Genome Biol.

